# Data for 3D reconstruction of the corticospinal tract in the wild-type and Semaphorin 6A knockout adult brain

**DOI:** 10.1016/j.dib.2019.103718

**Published:** 2019-03-07

**Authors:** Takuya Okada, Kazuko Keino-Masu, Fumikazu Suto, Kevin J. Mitchell, Masayuki Masu

**Affiliations:** a Department of Molecular Neurobiology, Faculty of Medicine, University of Tsukuba, 1-1-1 Tennodai, Tsukuba, Ibaraki, 305-8575, Japan; b Department of Ultrastructural Research, National Institute of Neuroscience, National Center of Neurology and Psychiatry, 4-1-1 Ogawa-Higashi, Kodaira, Tokyo, 187-8502, Japan; c Smurfit Institute of Genetics and Institute of Neuroscience, Trinity College Dublin, Ireland

## Abstract

The corticospinal tract (CST) has a complex and long trajectory throughout the brain. Semaphorin 6A (Sema6A), a member of the semaphorin family, is one of the important regulators of CST axon guidance. Previous studies have shown that *Sema6A* knockout (KO) mice have CST defects at the midbrain–hindbrain boundary and medulla [1]. However, the route of the aberrant fibers remained unknown. Therefore here, to track the trajectory of the abnormal fibers, 3D images of the CST in adult mice were reconstructed from serial brain sections stained with anti-PKCγ antibody. *Sema6A* mutant brains showed CST defects that were more complex and variable than previously thought. In addition, 3D analysis helped us to identify a few new patterns of abnormal fibers.

For more information about the data, please refer to an original research article, which has been recently published by Brain Research, “Remarkable complexity and variability of corticospinal tract defects in adult *Semaphorin 6A* knockout mice” [2].

**Table:** Specifications table

Subject area	*Biology*
More specific subject area	*Neuroscience*
Type of data	*Movie (.mp4), Table*
How data was acquired	*Immunohistochemistry, microscopy, image processing*
Data format	*Analyzed*
Experimental factors	*Brain sections were stained with anti-PKCγ antibody and photographed. 2D images were reconstructed into a 3D image using Imaris software.*
Experimental features	*Movies showing 3D images of the corticospinal tract in a wild-type mouse and three Sema6A knockout mice. In each movie, 360-degrees horizontal and vertical rotation are shown.*
Data source location	*Tsukuba, Japan*
Data accessibility	*Data is available from Mendeley Data.**https://data.mendeley.com/datasets/4t5wkg4pnh/draft?a=6c541991-599f-45f6-a4fc-3243b4c74bfd*
Related research article	*Okada, T., Keino-Masu, K., Suto, F., Mitchell, K.J., and Masu, M. Remarkable complexity and variability of corticospinal tract defects in adult Semaphorin 6A knockout mice. Brain Research, 1710, 209–219, 2019.*https://doi.org/10.1016/j.brainres.2018.12.041[2]

**Table:** 

**Value of the data** • These data describe the use of 3D reconstruction of the CST from serial antibody-stained sections to visualize the nerve trajectory throughout the mouse brain. • 3D reconstruction reveals the complex and variable CST defects in the Sema6A KO mice, which otherwise is difficult to intuitively notice using brain section analysis. • These data provide detailed information on the abnormalities of the neural circuits in the adult Sema6A KO mouse, which is valuable to understand the neurologic and psychologic deficits in the mutant mice [3] and human diseases.

## 1 Data

3D images of the CST trajectories throughout the adult mouse brains were reconstructed from serial sections stained with anti-PKCγ antibody. Each movie shows 360-degrees horizontal and vertical rotation of the 3D images of the CST. Images from one wild-type mouse (movies 1–3) and three *Sema6A* KO mice (movies 4–6, 7–9, 10–12) are shown.

The image of the wild-type mouse (shown in pale green) is inserted into the movies of *Sema6A* KO mice (pale blue). The characteristics of the datasets (dataset name, image attributes, file type, file size, and description of dataset) are summarized in Table 1.

**Table 1:** Dataset Characteristics

Dataset Name	Image Attributes	File Type	File Size
Movie 1	1280 × 720, pale green	MPEG-4 Movie (.mp4)	19.4 MB

Description: Movie showing a reconstructed 3D image of the CST of the wild-type mouse brain. The area from the cerebral peduncle to the dorsal funiculus is shown. This movie corresponds to the 3D reconstruction in Fig. 4A-A″ in Ref. [2]. The lateral (Figure 4A), ventral (Figure 4A′), and frontoventral (Figure 4A″) views are seen as the frames at 24, 40, and 38 seconds from the beginning of the movie, respectively.			

Dataset Name	Image Attributes	File Type	File Size
Movie 2	1280 × 720, pale green	MPEG-4 Movie (.mp4)	19.3 MB

Description: Movie showing a reconstructed 3D image of the CST of the wild-type mouse brain. The midbrain–hindbrain junction is shown. This movie corresponds to the 3D reconstruction in Fig. 4B-B″ in Ref. [2]. The lateral (Figure 4B), frontal (Figure 4B′), and frontoventral (Figure 4B″) views are seen as the frames at 25, 0, and 38 seconds from the beginning of the movie, respectively.			

Dataset Name	Image Attributes	File Type	File Size
Movie 3	1280 × 720, pale green	MPEG-4 Movie (.mp4)	19.4 MB

Description: Movie showing a reconstructed 3D image of the CST of the wild-type mouse brain. The pyramidal decussation is shown. This movie corresponds to the 3D reconstruction in Fig. 4C-C″ in Ref. [2]. The lateral (Figure 4C), frontal (Figure 4C′), and ventral (Figure 4C″) views are seen as the frames at 25, 0, and 40 seconds from the beginning of the movie, respectively.			

Dataset Name	Image Attributes	File Type	File Size
Movie 4	1280 × 720, pale blue	MPEG-4 Movie (.mp4)	19.4 MB

Description: Movie showing a reconstructed 3D image of the CST of the Sema6A KO #1 brain. The area from the cerebral peduncle to the dorsal funiculus is shown. This movie corresponds to the 3D reconstruction in Fig. 5A-A″ in Ref. [2]. The lateral (Figure 5A), ventral (Figure 5A′), and frontoventral (Figure 5A″) views are seen as the frames at 24, 40, and 36 seconds from the beginning of the movie, respectively. “Movie 1” of the wild-type mouse brain (pale green, inset) is shown as a reference.			

Dataset Name	Image Attributes	File Type	File Size
Movie 5	1280 × 720, pale blue	MPEG-4 Movie (.mp4)	19.3 MB

Description: Movie showing a reconstructed 3D image of the CST of the Sema6A KO #1 brain. The midbrain–hindbrain junction is shown. This movie corresponds to the 3D reconstruction in Fig. 5B-B″ in Ref. [2]. The lateral (Figure 5B), frontal (Figure 5B′), and frontoventral (Figure 5B″) views are seen as the frames at 24, 0, and 37 seconds from the beginning of the movie, respectively. “Movie 2” of the wild-type mouse brain (pale green, inset) is shown as a reference.			

Dataset Name	Image Attributes	File Type	File Size
Movie 6	1280 × 720, pale blue	MPEG-4 Movie (.mp4)	19.3 MB

Description: Movie showing a reconstructed 3D image of the CST of the Sema6A KO #1 brain. The pyramidal decussation is shown. This movie corresponds to the 3D reconstruction in Fig. 5C-C″ in Ref. [2]. The lateral (Figure 5C), frontal (Figure 5C′), and ventral (Figure 5C″) views are seen as the frames at 24, 0, and 40 seconds from the beginning of the movie, respectively. “Movie 3” of the wild-type mouse brain (pale green, inset) is shown as a reference.			

Dataset Name	Image Attributes	File Type	File Size
Movie 7	1280 × 720, pale blue	MPEG-4 Movie (.mp4)	19.3 MB

Description: Movie showing a reconstructed 3D image of the CST of the Sema6A KO #2 brain. The area from the cerebral peduncle to the dorsal funiculus is shown. This movie corresponds to the 3D reconstruction in Fig. 6A-A″ in Ref. [2]. The lateral (Figure 6A), ventral (Figure 6A′), and frontoventral (Figure 6A″) views are seen as the frames at 24, 41, and 36 seconds from the beginning of the movie, respectively. “Movie 1” of the wild-type mouse brain (pale green, inset) is shown as a reference.			

Dataset Name	Image Attributes	File Type	File Size
Movie 8	1280 × 720, pale blue	MPEG-4 Movie (.mp4)	19.3 MB

Description: Movie showing a reconstructed 3D image of the CST of the Sema6A KO #2 brain. The midbrain–hindbrain junction is shown. This movie corresponds to the 3D reconstruction in Fig. 6B-B″ in Ref. [2]. The lateral (Figure 6B), frontal (Figure 6B′), and frontoventral (Figure 6B″) views are seen as the frames at 24, 0, and 37 seconds from the beginning of the movie, respectively. “Movie 2” of the wild-type mouse brain (pale green, inset) is shown as a reference.			

Dataset Name	Image Attributes	File Type	File Size
Movie 9	1280 × 720, pale blue	MPEG-4 Movie (.mp4)	19.4 MB

Description: Movie showing a reconstructed 3D image of the CST of the Sema6A KO #2 brain. The pyramidal decussation is shown. This movie corresponds to the 3D reconstruction in Fig. 6C-C″ in Ref. [2]. The lateral (Figure 6C), frontal (Figure 6C′), and ventral (Figure 6C″) views are seen as the frames at 25, 0, and 40 seconds from the beginning of the movie, respectively. “Movie 3” of the wild-type mouse brain (pale green, inset) is shown as a reference.			

Dataset Name	Image Attributes	File Type	File Size
Movie 10	1280 × 720, pale blue	MPEG-4 Movie (.mp4)	19.4 MB

Description: Movie showing a reconstructed 3D image of the CST of the Sema6A KO #3 brain. The area from the cerebral peduncle to the dorsal funiculus is shown. This movie corresponds to the 3D reconstruction in Fig. 7A-A″ in Ref. [2]. The lateral (Figure 7A), ventral (Figure 7A′), and frontoventral (Figure 7A″) views are seen as the frames at 24, 40, and 37 seconds from the beginning of the movie, respectively. “Movie 1” of the wild-type mouse brain (pale green, inset) is shown as a reference.			

Dataset Name	Image Attributes	File Type	File Size
Movie 11	1280 × 720, pale blue	MPEG-4 Movie (.mp4)	19.4 MB

Description: Movie showing a reconstructed 3D image of the CST of the Sema6A KO #3 brain. The midbrain–hindbrain junction is shown. This movie corresponds to the 3D reconstruction in Fig. 7B-B″ in Ref. [2]. The lateral (Figure 7B), frontal (Figure 7B′), and frontoventral (Figure 7B″) views are seen as the frames at 24, 0, and 37 seconds from the beginning of the movie, respectively. “Movie 2” of the wild-type mouse brain (pale green, inset) is shown as a reference.			

Dataset Name	Image Attributes	File Type	File Size
Movie 12	1280 × 720, pale blue	MPEG-4 Movie (.mp4)	19.4 MB

Description: Movie showing a reconstructed 3D image of the CST of the Sema6A KO #3 brain. The pyramidal decussation is shown. This movie corresponds to the 3D reconstruction in Fig. 7C-C″ in Ref. [2]. The lateral (Figure 7C), frontal (Figure 7C′), and ventral (Figure 7C″) views are seen as the frames at 25, 0, and 40 seconds from the beginning of the movie, respectively. “Movie 3” of the wild-type mouse brain (pale green, inset) is shown as a reference.			

## 2 Experimental design, materials and methods

### 2.1 Animals

The *Sema6A* KO mice were described previously [1], [3]. All animal experiments were approved and performed according to the guidelines of the Animal Care and Use Committees of the University of Tsukuba and the National Institute of Neuroscience, National Center of Neurology and Psychiatry.

### 2.2 Immunohistochemistry

Cryostat sections (50 μm) of paraformaldehyde-fixed adult mouse brains were dehydrated, treated with 3% H_2_O_2_ in 80% methanol, 20% dimethyl sulfoxide (DMSO) for 30 min, rehydrated, and incubated with anti-PKCγ antibody (1:200; Frontier Institute, Hokkaido, Japan) at 4 °C twice overnight. After incubation with avidin-biotin complex (Vectastain Elite ABC kit; Vector Laboratories, Burlingame, CA, USA) for 30 min and washing, the sections were incubated with 3,3′-diaminobenzidine (DAB; Vector Laboratories) for 10 min. The sections were mounted on MAS-coated glass slides (Matsunami Glass Industries, Osaka, Japan) and covered with coverslips using Fluoromount-G (SouthernBiotech, Birmingham, AL, USA). Bright field images were obtained using a fluorescence microscope (BZ-8000; Keyence, Osaka, Japan).

### 2.3 3D reconstruction

The 2D images of serial stained sections were aligned using AutoAligner software (Bitplane, Zürich, Switzerland). The signals in the thalamus, hypothalamus, brainstem, and spinal cord were extracted using Photoshop software (Adobe Systems, San Jose, CA, USA). Stacks of the aligned images were imported into Imaris software (Bitplane) and transformed into 3D images.
